# Factors Influencing Satisfaction With Online Learning During COVID-19 Crisis by Undergraduate Medical Students From King Saud bin Abdulaziz University for Health Sciences (KSAUHS), Riyadh

**DOI:** 10.7759/cureus.41672

**Published:** 2023-07-10

**Authors:** Sameerah Alsomali, Ziad Mandourah, Yasser Almashari, Sulaiman Alayed, Yazan Alqozi, Abdulrahman Alharthi

**Affiliations:** 1 Emergency Medicine, King Abdulaziz Medical City Riyadh, Riyadh, SAU; 2 Emergency Medicine, College of Applied Medical Sciences, King Saud bin Abdulaziz University for Health Sciences, Riyadh, SAU; 3 Medicine and Surgery, College of Medicine, King Saud Bin Abdulaziz University for Health Sciences, Riyadh, SAU; 4 Medicine, College of Medicine, King Saud bin Abdulaziz University for Health Sciences, Riyadh, SAU

**Keywords:** satisfaction, factors, questionnaire, covid-19, online learning

## Abstract

Background

During the coronavirus 2019 (COVID-19) pandemic, Saudi Arabia implemented strict measures to prevent the entry and spread of the virus and to minimize its burden on society, including the use of online education as an alternative to traditional classroom learning. This study surveyed medical students from King Abdulaziz bin Saud University for Health Sciences-Riyadh to evaluate the factors affecting their satisfaction and experience with online learning.

Methods

This cross-sectional study was conducted from March to May 2021, using consecutive sampling and a population of approximately 700 medical students from King Saud bin Abdulaziz University of Health Sciences. This study was carried out through an online questionnaire.

Results

The survey received 277 responses with a response rate of 40%. About 15.3% of respondents believed that online learning productivity was poor, and 18.9% found it below average; however, 21.7% rated it as excellent. The level of anxiety while studying at home was rated as fine by 32.7%, while 21% felt very anxious, and the level of distraction was reported to be high, by 23.1%. Additionally, 64.4% of respondents preferred more online courses even after the COVID-19 crisis. Overall, 29.2% of respondents rated online learning as excellent, while 12.5% rated it as poor. We found a significant positive correlation between feeling isolated while studying at home and feeling anxious (r=0.618; p<0.001) and a significant positive correlation between productivity during online learning and the impact on grades (r=0.495; p<0.001).

Conclusion

This study found that online learning had benefits but also negatively impacted academic performance and mental health, highlighting the need for tailored support services. Further research is recommended, and medical students should have more theoretical online classes while keeping practical sessions on-site.

## Introduction

Coronavirus is a group of RNA viruses that cause respiratory infections, ranging from a common cold to severe illnesses like Middle East Respiratory Syndrome (MERS) and Severe Acute Respiratory Syndrome (SARS) [1]. COVID-19 is the most recent coronavirus strain discovered in December 2019, which has since spread globally, causing a pandemic [2]. The outbreak has affected various aspects of life, including culture, ecology, and society, resulting in lockdowns and restrictions [3,4]. Most governments implemented social distancing measures to limit the spread of COVID-19. Saudi Arabia has taken strict measures to prevent the entry of the virus and reduce its impact [5]. Online education is one of the measures implemented to limit exposure for students and faculty members in Saudi Arabia [6,7].

Online education refers to 100% virtual courses offered by postsecondary institutions as an alternative to traditional classroom-based learning. It allows students and teachers to learn at their own pace and schedule [8]. In response to the COVID-19 pandemic, many educational institutions worldwide have closed, resulting in a peak of 1.598 billion learners from 194 countries required to stay home as of Apr 1, 2020. Studies suggest that online education faces challenges for the teaching faculty, such as time-consuming lesson planning, inadequate technical support, and a lack of online teaching skills among educators [9]. Additionally, students without reliable internet access or computers/laptops struggle to participate in e-learning, accentuating the learning gap for students from different income brackets [10]. For instance, while 95% of students in Switzerland, Norway, and Austria have a computer for schoolwork, only 34% in Indonesia do, according to OECD data [11]. Furthermore, students in certain parts of the world, such as India, Iraq, Iran, and Syria, experience poor internet connectivity [12].

Due to the recent inclusion of online learning in the Kingdom of Saudi Arabia, there is limited research on student attitudes and interactions toward this teaching method. A study conducted on King Abdul Aziz students revealed that they faced significant obstacles to taking e-learning courses, including material and financial barriers, technical difficulties, organizational and administrative hurdles, as well as systemic obstacles related to the education system [13]. There is currently no research on online learning at King Saud bin Abdul-Aziz University for Health Sciences in Riyadh (KSAUHS-R), and most existing studies have not explored students' emotional reactions (e.g., acceptance, related beliefs, and the perceived ease of use) to this learning method, particularly during the pandemic. Furthermore, research tends to lack specificity toward particular subsets of students, such as medical, business, or engineering students, who may offer unique perspectives on the topic. Therefore, this study aimed to investigate the factors influencing the process of education during the pandemic. Moreover, we aimed to evaluate the perspectives of the students towards online education compared to traditional education.

## Materials and methods

Study design, area, and settings

A cross-sectional study was conducted from March 7th, 2021, to May 4th, 2021, based on a questionnaire administered to students at KSAUHS-Riyadh. The study was approved by the ethical committee of the KSAUHS-Riyadh (IRB: SP21R/022/02). Prior to participating in the study, the participants were required to provide written consent, indicating their understanding of the study objectives and the measures taken to protect their privacy.

Inclusion and exclusion criteria

The study included undergraduate students from KSAUHS-Riyadh who attended during the year 2020 and agreed to participate by providing written consent through the questionnaire. The exclusion criteria were postgraduate students and those who had already graduated. Undergraduate medical students were selected for the study using the consecutive sampling technique, where all subjects who met the inclusion criteria were chosen.

Sample size calculation

The total population under consideration was 700, and the sample size calculation was performed using SurveyMonkey Online Calculator (https://www.surveymonkey.com/mp/sample-size-calculator/). This calculator employs the following formula for determining the sample size (n): n = N * X / (X + N - 1), where X = Zα/2^2 * p(1-p) / MOE^2. In this formula, Zα/2 represents the critical value of the normal distribution at α/2 (for example, with a 95% confidence level, α is 0.05, and the critical value is 1.96), MOE denotes the margin of error (0.05), p signifies the sample proportion (50%), and N stands for the population size. Based on these inputs, the estimated sample size was 249.

Data collection process and tools

In the development of our survey, we followed a meticulous, multi-step process to ensure its robustness and relevance to our research objectives. Firstly, our team diligently crafted the survey, utilizing our collective expertise and prior knowledge. Each question was designed with careful consideration of its relevance to the study's goals to ensure the effective collection of meaningful and accurate data. In the second phase of survey development, we sought external validation by engaging three experienced consultants. These individuals, possessing backgrounds in both medical education and psychology, were selected due to their professional expertise and their ability to evaluate the survey's content from multidisciplinary perspectives. They meticulously reviewed our survey and provided insightful recommendations and corrections. This step aimed to enhance the survey's validity and reliability, as well as to reduce any potential bias. Subsequently, to further refine the survey's structure and improve its psychometric properties, we incorporated the expert opinion of a seasoned psychometrician. The psychometrician meticulously evaluated the survey for potential errors, inconsistencies, or bias in question phrasing. Their input was invaluable in enhancing the accuracy and reliability of our tool. Following these initial development and review stages, we then conducted a pilot test of the survey with a group of 20 participants. This initial group was chosen to reflect the broader population we aimed to study, thereby providing us with an opportunity to assess the survey's functionality and effectiveness in a real-world setting. Once the data from this preliminary test were collected, we performed a thorough analysis to identify potential issues, gaps, or confounding factors. We adjusted the survey accordingly based on the feedback and results from this pilot testing. The modifications were carefully made to ensure that the final version of the survey would yield reliable and valid data when deployed in our full-scale research study. Data were collected via online questionnaires sent to undergraduate students at KSAUHS-Riyadh. Online Google Form was used to manage the responses of included participants. The questionnaire consisted of 21 Likert-scale questions designed to evaluate various aspects of online learning. These aspects included education productivity, efficiency, impact on grades, influence on study habits, degree of isolation, anxiety levels, and accessibility to internet connections. Throughout the study, subjects were notified of the measures taken to protect their confidentiality. Security protocols, such as password protection, were utilized when transferring participant data to our central server. Any information that may identify or expose a participant, including names and telephone numbers, was removed, and code words were provided instead of identifying information. Productivity was defined as the ability of the students to effectively manage their time, efforts, and resources to achieve their learning goals. Efficiency was defined as the ability of the students to achieve desired learning outcomes with a minimum of wasted effort or expense.

Data analysis

Data entry and analysis were conducted using the SPSS (IBM Corp. Released 2013. IBM SPSS Statistics for Windows, Version 22.0. Armonk, NY: IBM Corp). Descriptive statistics, such as frequencies and percentages, were used for ordinal variables, and Spearman's rho was utilized for testing. A p-value less than 0.05 was considered statistically significant.

## Results

**Figure 1:**
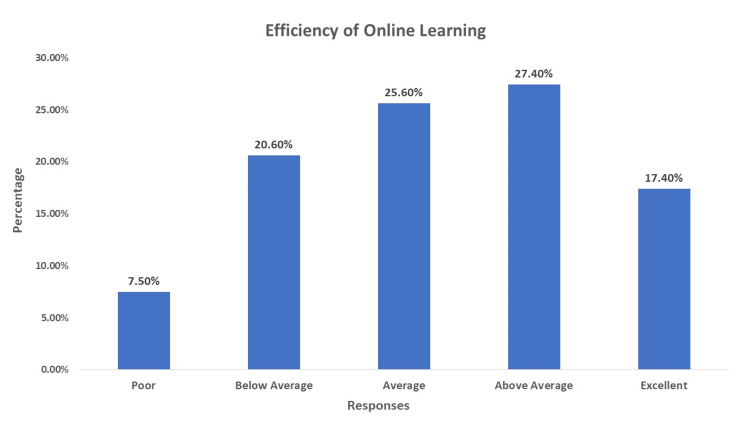
Efficiency of Online Learning This figure shows included participants' responses toward the efficiency of online learning.

The survey received 277 responses from a total of 700 students, resulting in a response rate of 40%. Most respondents (n=217, 78.34%) were aged between 18 and 21. Regarding productivity, we found that 43 (15.3%) of respondents believed online learning was poor, while 53 (18.9%) found it below average. However, 61 (21.7%) of respondents rated it as excellent. Meanwhile, 21 (7.5%) rated the efficiency of online learning as poor, 77 (27.4%) rated it as above average, and another 49 (17.4%) rated it as excellent, as shown in Figure 1.

**Figure 2:**
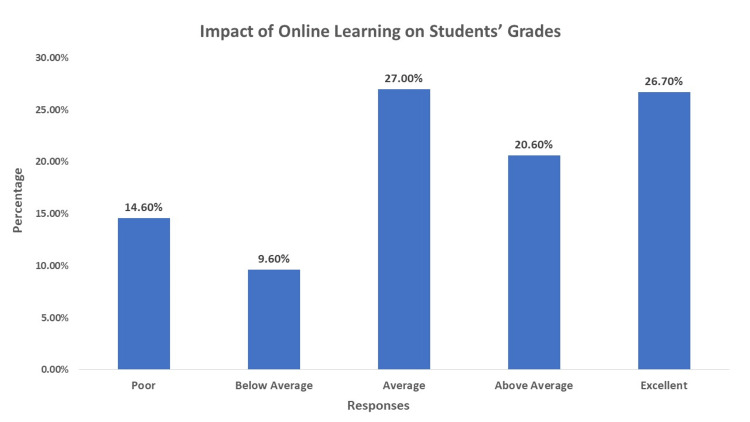
Impact of Online Learning on Students' Grades This figure shows the responses of included participants toward the impact of online learning on their grades.

Regarding the impact of online learning on students' grades, 41 (14.6%) of respondents rated it as poor, while 75 (26.7%) rated it as excellent, as shown in Figure 2.

In terms of the level of anxiety and distraction experienced by students while studying at home, 92 (32.7%) of respondents rated it as fine, while 59 (21%) felt very anxious. The level of distraction at home was ranked as high by 65 (23.1%) respondents. Most respondents (n= 181, 64.4%) preferred having more online courses even after resolving the COVID-19 crisis.

Regarding the ability of students to study during online learning, 75 (26.7%) of respondents felt significantly impacted by the pandemic, while 36 (12.8%) were barely affected. The level of isolation experienced by students during online learning was rated as occasionally by 51 (18.1%) of respondents, and 98 (34.9%) felt isolated. Concerning the impact of online learning on clinical and practical skills, 87 (31%) of respondents felt significantly impacted, while 8 (2.8%) were barely affected. Most respondents (n=213, 75.8%) did not build any connections with their colleagues, as shown in Table 1.

**Table 1:** Participants' responses Data were presented as frequencies and percentages.

Themes		N (%)
Productivity	Poor	43 (15.3%)
	Below Average	53 (18.9%)
	Average	50 (17.8%)
	Above Average	70 (24.9%)
	Excellent	61 (21.7%)
Studying at home	Fine	92 (32.7%)
	Barely anxious	33 (11.7%)
	Occasionally	50 (17.8%)
	Frequently	43 (15.3%)
	Very Anxious	59 (21%)
Would you like to have more online courses after the resolution of the COVID-19 crisis?	Yes	181 (64.4%)
	No	96 (34.2%)
Were you able to study during online learning?	Barely Impacted	36 (12.8%)
	Slightly	26 (9.3%)
	Occasionally	85 (30.2%)
	Frequently	55 (19.6%)
	Greatly impacted	75 (26.7%)
Level of distraction at home	Low	48 (17.1%)
	Moderately Low	43 (15.3%)
	Moderate	54 (19.2%)
	Moderately High	67 (23.8%)
	High	65 (23.1%)
Level of Isolation	Not Isolated	46 (16.4%)
	Slightly	26 (9.3%)
	Occasionally	51 (18.1%)
	Frequently	56 (19.9%)
	Isolated	98 (34.9%)
Clinical and practical skills	Barely Impacted	8 (2.8%)
	Slightly	44 (15.7%)
	Occasionally	77 (27.4%)
	Frequently	60 (21.4%)
	Greatly impacted	87 (31%)
Have you built any connections with your colleagues during online learning?	No	213 (75.8%)
	Yes	64 (22.8%)

**Figure 3:**
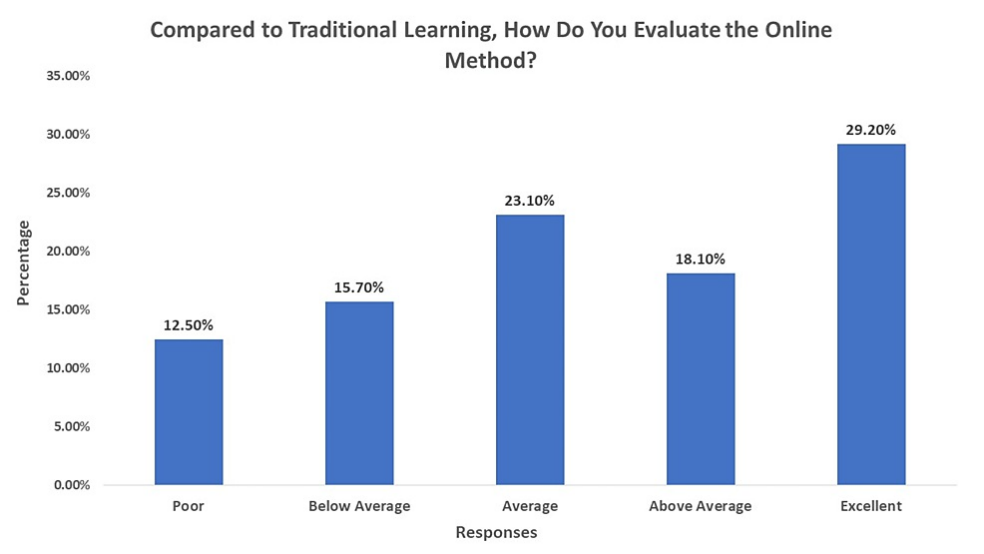
Comparison between Traditional Learning and Online Learning This figure shows the participants' responses to how they evaluate online learning compared to traditional education.

Overall, the evaluation of online learning compared to traditional education was mixed, with 29.2% of respondents rating it as excellent, while 12.5% rated it as poor, as shown in Figure 3.

Analytical analysis

There was a significant positive correlation between feeling isolated while studying at home and feeling anxious, with a moderate correlation coefficient of (r=0.618; p<0.001). Similarly, the results also indicate a significant positive correlation between productivity during online learning and the impact of online learning on grades, with a moderate correlation coefficient of (r=0.495; p<0.001).

## Discussion

This study aimed to investigate the perceptions and experiences of undergraduate students at KSAUHS-Riyadh towards online learning during the COVID-19 pandemic. The study results showed disparate responses from the respondents, with some reporting positive experiences and others reporting negative experiences. The response rate of 40% was lower than expected, which may have implications for the generalizability of the findings. However, the sample size of 277 students was sufficient to provide a representative view of the population under study. The relatively low response rate of 40% in our study may be attributed to the high-stress environment during the COVID-19 pandemic, increased online activities, survey fatigue, and potential privacy concerns, which could have influenced the willingness or ability of students to participate in this online questionnaire.

Regarding productivity, the study found that 34.2% of the students rated online learning as poor or below average. This highlights the challenges students face in adjusting to online learning, which may have been due to a lack of access to technology, poor internet connectivity, and difficulty adapting to a new learning environment. Khalil et al. (2020) analyzed the attitudes and preferences of undergraduate medical students in Saudi Arabia toward synchronized online learning [14]. They found that students who assented to the online learning modules were motivated by the benefit of mastering content in less time compared to traditional classroom learning, utilizing updated educational technologies that promote active and student-centered learning [14]. Students found that online learning allowed them to control their educational needs constructively and provided structured guidance for self-directed learning [14]. The study also found that while online learning was more productive in preclinical subjects, clinical experience, and human interaction were crucial for the practice of medicine [14]. Several studies also found that preclinical students preferred online learning for their future academic years [15,16]. While clinical experience and human interaction are essential for the practice of medicine, incorporating virtual simulation technologies and computer-based models into online learning can provide clinical students with controlled opportunities to practice rare and critical events safely without risking patients' well-being [17].

The barriers to online learning included technical insufficiency [18-20], individual characteristics of students [21,22], and quality assurance issues in the implementation of online education by the institution [14]. Khashaba et al. (2022) showed that IT and technical skills were strongly linked to effective communication, convenient learning, and online learning technology [23]. These findings indicate that the productivity of students and the benefits of online learning could be affected by several factors, including IT and technical skills, poor internet connectivity, and individual characteristics of students.

The study also found that a significant percentage of students experienced anxiety and felt isolated while studying at home, which may have had a negative impact on their mental health and academic performance. This finding is consistent with previous studies that have reported the adverse effects of social isolation and anxiety on academic performance. Pelucio and his colleagues (2022) showed that among 152 online learning students during the COVID-19 pandemic, most participants had moderate anxiety levels, with no significant differences between males and females. The younger students reported higher anxiety levels than older students, and female students with limited social contact reported higher levels of depression [24]. Nearly half of the students in one study exhibited anxiety ranging from mild to severe, with females showing higher anxiety scores [25]. In contrast, Saddik et al. (2020) showed that female students scored significantly higher than males on measures of depression, anxiety, and stress as the pandemic progressed [26], and longitudinal studies of college students have shown a significant increase in depression and anxiety levels compared to pre-pandemic levels [27].

Interestingly, our study found a significant positive correlation between productivity during online learning and the positive impact of online learning on grades. This suggests that students who perceived online education as positively impacting their grades could be more productive in their studies. This finding has important implications for the design and implementation of online learning programs, as it highlights the need to focus on strategies that can improve students' perception of the impact of online learning on their academic performance.

We acknowledge that our study has some limitations, including the low response rate and the absence of detailed analysis based on the student's characteristics and types of online courses. Additionally, this survey did not assess the accessibility of the Internet and computer devices in the studied population. Therefore, further studies are required to cover this gap and provide more understanding of the potential factors that may influence the online learning process.

## Conclusions

This study provides valuable insights into the experiences of undergraduate students at KSAUHS-Riyadh during the COVID-19 pandemic. The study's findings suggest that the majority of students prefer online learning over traditional learning; however, others have faced challenges that have negatively impacted their academic performance and mental health. The study's results can be used to inform the design and implementation of online learning programs and support services that address the needs of students during times of crisis. However, further research is needed to explore these issues in more detail and develop effective strategies to support students during the pandemic and beyond. We recommend including more theoretical online classes for medical students, emphasizing keeping practical sessions on-site.
